# Teduglutide in amyloidosis‐associated intestinal failure

**DOI:** 10.1002/ccr3.7653

**Published:** 2023-08-17

**Authors:** Clara Luhn, Hermine Agis, Elisabeth Hütterer, Ingrid Simonitsch‐Klupp, Christopher Dawoud, Anton Stift, Felix Harpain

**Affiliations:** ^1^ Division of Visceral Surgery, Department of General Surgery Medical University Vienna Vienna Austria; ^2^ Division of Hematology and Hemostaseology, Department of Internal Medicine I Medical University of Vienna Vienna Austria; ^3^ Division of Oncology, Department of Internal Medicine I Medical University of Vienna Vienna Austria; ^4^ Department of Pathology Medical University of Vienna Vienna Austria

**Keywords:** amyloidosis, enteral autonomy, glucagon‐like peptide‐2, intestinal failure, teduglutide

## Abstract

Amyloidosis is a heterogeneous disease characterized by tissue deposition of abnormally folded fibrillary proteins that can manifest itself by a wide variety of symptoms depending on the affected organs. GI involvement among amyloidosis patients is common. Its clinical manifestation often presents with nonspecific symptoms such as weight loss, diarrhea, and malabsorption. With no specific treatment existing for GI amyloidosis, therapy focuses on impeding amyloid deposition and managing the patients' symptoms with supportive measures. Here, we present an AL‐amyloidosis patient with GI involvement and intestinal failure (IF) who was successfully treated with the glucagon‐like peptide‐2 (GLP‐2) analogue teduglutide. Over the course of treatment with teduglutide, the patient was able to achieve independence from parenteral nutrition and experienced a significant improvement in quality of life (QoL) as stool frequency and consistency improved, urinary output was stabilized and body weight as well as body composition improved over the course of teduglutide therapy. With no longer being exposed to the burden and associated risks of parenteral nutrition, we were able to reduce the potential morbidity and mortality rate as well as to improve the patient's overall QoL. Intestinal tissue biopsy workup revealed a histopathological correlate for the clinical response; Congo‐Red‐positive intestinal depositions almost completely disappeared within 6 months of teduglutide therapy. Implementing intestinotrophic GLP‐2 analogue teduglutide may enrich the spectrum of treatment options for amyloidosis patients with IF who are dependent on parenteral support.

## Graphical abstract

Graphical Abstract

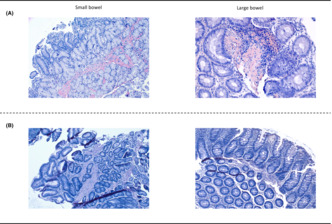

## 1 BACKGROUND

Amyloidosis is a chronic progressive disease in which misfolded, insoluble as well as digestion‐resistant proteins accumulate in various tissues causing severe structural and functional organ damage.^1^, ^2^ As the body is unable to eliminate these accumulating proteins, they permanently disrupt the enzyme function and cell metabolism and thereby impair the function of the organs where they have accumulated. The deposition of these insoluble fibrillary proteins, called amyloids, may affect any tissue or organ. However, the heart, kidneys, liver, nervous system, and gastrointestinal (GI) tract are most often affected.^3^, ^4^ Several subforms of amyloidosis may be classified based on the involved amyloidogenic protein, the associated clinical syndromes, and the extent of organ system involvement.^3^

Within generalized amyloidosis, the GI tract is often affected by amyloids primarily deposited in the small intestine.^5^ The misfolded proteins mostly appear in the muscularis mucosae and stromal tissue affecting the vasculature, nerves, and nervous plexuses close by leading to impaired intrinsic peristalsis and absorption.^6^, ^7^ Typical symptoms are constipation, alternating constipation and diarrhea, chronic diarrhea, abdominal pain, and malabsorption leading to severe weight loss since enteral nutrient and fluid supply can no longer sufficiently be utilized.^7^, ^8^, ^9^ In addition, GI tract involvement also significantly affects the overall survival in amyloidosis patients: Lim et al.^10^ observed in a prospective cohort study of AL‐amyloidosis patients a 15.84 months survival in patients without GI involvement and 7.95 months with GI involvement.

Intestinal failure (IF), as the worst consequence of malabsorption, is not only associated with increased morbidity and mortality but also associated with poor quality of life (QoL).^11^, ^12^

IF in short bowel syndrome (SBS) patients is caused by surgical bowel resection resulting in patients being unable to meet nutritional needs for enteral autonomy and are therefore dependent on parental support (PS).^13^

A significant impact in treating IF was achieved with the glucagon‐like peptide‐2 (GLP‐2) analogue teduglutide.^14^ GLP‐2 is secreted from the enteroendocrine L‐cells of the terminal ileum and colon and was discovered and described as a key factor in intestinal rehabilitation and growth.^15^ GLP‐2 stimulates the growth of the intestinal mucosa through stimulation of crypt cell proliferation and inhibition of enterocyte apoptosis.^16^, ^17^ Additionally, GLP‐2 impedes gastric emptying and acid secretion, stimulates intestinal blood flow, and increases the intestinal barrier function.^15^ The drug is approved for patients suffering from SBS with chronic IF and enhances the intestinal absorption of fluids and nutrients and, therefore, can reduce the patients' need for parenteral support.^18^

To the best of the authors' knowledge, this case report is first to present the use of teduglutide in treating AL‐amyloidosis‐associated IF.

## 2 CASE REPORT

In this case report, we present a 45‐year‐old male patient with AL‐amyloidosis with renal and enteric involvement (iFLC [involved free light chain] 103.2 mg/L, dFLC [difference free Light chain] 71.1 mg/L, kappa/lambda ratio 0.31). The patient was first presented to our hospital after the initial diagnosis of renal AL‐amyloidosis of the kidneys was made and transferred with suspicion of GI involvement.

**FIGURE 1:**
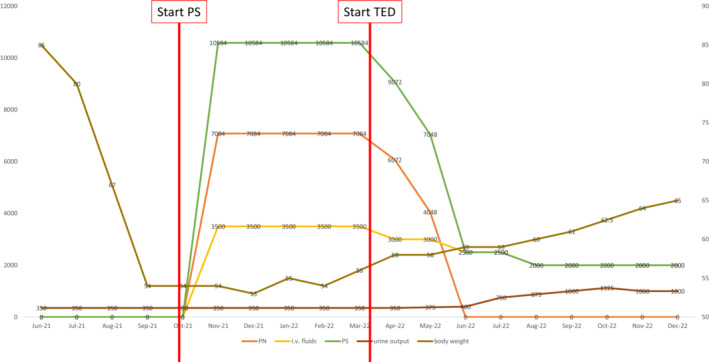
Clinical response to teduglutide therapy. Left *y*‐axis depicting parenteral support volume in milliliter (mL), right *y*‐axis body weight in kilogram [kg]. i.v., intravenous; PN, parenteral nutrition; PS, parenteral support; TED, teduglutide.

The patient presented to an outside hospital with progressive edema of the lower extremities, shortness of breath, massive diarrhea, and body weight loss of unknown origin. A nephrotic range proteinuria (Palladini Stage II^19^ with a protein/creatinine ratio 24,530 mg/g, albumin/creatinine ratio 22,696 mg/g, eGFR of >50 mL/min per 1.73 m^2^) prompted a kidney biopsy with the histopathologic workup clearly revealing an AL‐amyloidosis with glomerular deposition of lambda light chains. After having already lost 10 kg within 6 months, the patient was introduced to our clinical department of hematology and hemostaseology and treated interdisciplinary with our IF unit (see Figure 1 for body weight course).

**FIGURE 2:**
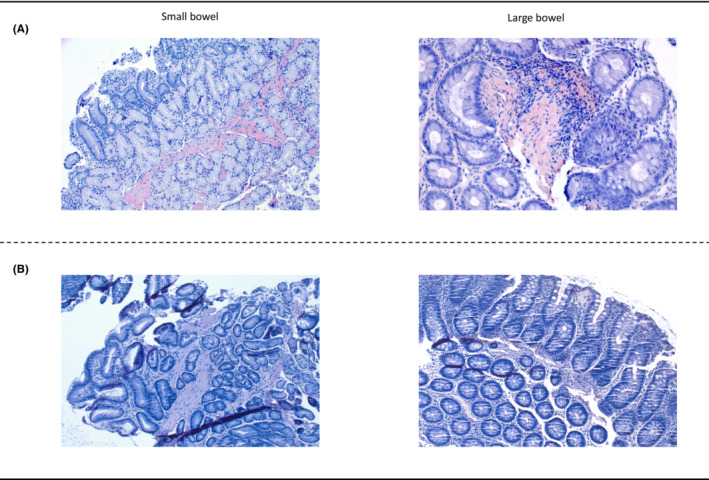
Congo‐Red staining before (A) and after 6 months (B) of teduglutide therapy. Panel (A) depicting Congo‐Red staining of small and large bowel biopsies showing conspicuous deposition of intensely stained homogeneous amorphous fibrillary material in the lamina propria (LP; focal green autofluorescence was noted in polarized light, not shown). Panel (B) revealed Congo‐Red staining of both small‐ and large bowel biopsies after 6 months of treatment, demonstrating only single fibers (small bowel) with weak specific red staining within the LP; the biopsy of the large bowel showed no Congo‐Red positive depositions.

The patient suffered from massive diarrhea (8–12 bowel movements per day, Bristol stool scale 6–7^20^), malabsorption, fecal incontinence, leg edema, hypotonic blood pressure, and disorientation due to his physical weakness. A bone marrow biopsy showed approximately 20% atypical plasma cells with an aberrant immunophenotype (CD20+/Cyclin‐D1+/CD117+/Lambda+) and vascular AL‐amyloidosis. An initial colonoscopy with random intestinal biopsies histologically confirmed GI involvement by AL‐amyloidosis (see Figure 2). In addition to the nephrotic syndrome that led to an extreme protein loss, the severe diarrhea increased the patient's protein deficiency. Furthermore, the diagnostic workup revealed no cardiac involvement by amyloidosis, staging the patient at Mayo 2012 stage 0 (Troponin T 0.014 ng/dL, proBNP 223 pg/mL, dFLC 71.1 mg/L).^21^

Symptomatic therapy in terms of antidiarrheal and antisecretory agents and daily albumin infusions was performed. Additionally, the patient received tight‐meshed counseling through our SBS unit dietary program. Unfortunately, however, a satisfying improvement of the overall medical condition could not be achieved by it.

The underlying plasma cell dyscrasia was treated with the monoclonal antibody daratumumab and the proteasome inhibitor bortezomib: The patient received an induction therapy consisting of three cycles of daratumumab and dexamethasone and nine cycles of daratumumab, bortezomib and dexamethasone followed by a maintenance therapy of daratumumab and dexamethasone. With that treatment, the patient revealed a good hematologic response (iFLC of 31.7 mg/L, dFLC of 7.9 mg/L). Nevertheless, in terms of organ therapy response, we observed a disease progression: although an improvement of the protein/creatinine ratio and albumin/creatinine ratio with 4459 and 3380 mg/g, respectively, could be achieved, renal function deteriorated with an over 25% increase of serum creatinine after nine cycles of therapy (from 1.12 to 2.51 mg/dL with a concomitant decrease of the glomerular filtration rate from 63.63 to 28.07 mL/min per 1.73 m^2^).^22^ We waived periodic 24 h urine protein measurements as those were biased anyway by the massive volume losses caused by the diarrhea.

In addition, the patient continued to be severely malnourished, with a body mass index of 19 kg/m^2^ and laboratory tests revealing his malnourishment. He continued to suffer from severe diarrhea resulting in energy and fluid loss subsequently making him dependent on PS. He was started on a parenteral nutrition (PN; 900 kcal/day) and intravenous fluids with additional water‐soluble and fat‐soluble vitamins, trace elements, and albumin (20 g) on a daily basis (see Figure 2 for the course of PS). With the PS, the excessive weight loss of the patient (now already over 30 kg in 4 months) could be stopped. Although he was able to maintain his weight with the offered PS, gaining body weight was not possible and his general physical weakness was obvious.

Considering the patient's course and the prognosis of the disease and his poor QoL, which was severely affected by the diarrhea, the need for PS, and his risk of central line‐associated bloodstream infections (CLABSI) and sepsis, the option of off‐label treatment with teduglutide was discussed within an interdisciplinary team. The possibility of achieving enteral autonomy through teduglutide therapy and adjusting him to an optimal diet without the disadvantages of PS was presented to the patient who agreed to a therapy attempt with teduglutide.

At the beginning of the treatment, the patient received an adjusted dose of teduglutide due to his renal function impairment. Nevertheless, the patient showed an excellent response and tolerance to teduglutide so that the dose could be increased to the full, body weight‐adjusted dose with improvement of the renal function after 3 months.

Throughout treatment with teduglutide, the patient's dependency on PN was eliminated (see Figure 1). The patient's leg edema also regressed and his physical condition improved due to his body weight gain. In addition, we also observed a symptomatic response as his daily bowel movements were reduced down to two to four per day (with an improved stool consistency, Bristol stool scale 4–5) with a stable daily urine output of around 1000 mL per day (see Figure 1 for urine output over time). The excellent clinical course under teduglutide therapy prompted a follow‐up colonoscopy. Analysis of small and large bowel tissue biopsies revealed an almost complete disappearance of the Congo‐Red positive material as a potential histopathological correlate to the good clinical response (see Figure 2).

## 3 DISCUSSION

This case report presents an AL‐amyloidosis patient with severe enteric involvement leading to IF who was treated with the GLP‐2 analogue teduglutide and thereby was able to discontinue PN and simultaneously achieve a good nutritional status. Histological workup revealed that intestinal amyloid deposits nearly disappeared after 6 months of teduglutide therapy.

Amyloidosis is a poor prognosis disease and is known to manifest itself in a wide array of symptoms depending on the involved organ with most patients having several organ systems involved at presentation.^4^ The GI tract is often affected in patients with amyloidosis and denotes a significant risk factor for poor prognosis as it nearly halves the median survival compared to amyloidosis patients with an unaffected intestine.^7^, ^10^, ^23^

GI involvement also played a dominant role in the course of the disease in the here presented case. Unable to be sufficiently alleviated by symptomatic therapy in terms of antidiarrheal and antisecretory agents and daily albumin infusions the general health status worsened over the initial course of therapy. Although the amyloidosis‐specific therapy achieved a good hematologic response, organ response (GI and renal) was poor. With decreasing physical strength and malnourishment and a significant body weight loss, PS was an unavoidable measure in the course of treatment leading to a stabilization of the health status. However, with PS it was impossible to sufficiently improve the patients' symptoms and enable a significant body weight gain. Furthermore, due to PS the patient was now also exposed to new risks such as CLABSI, central thrombosis, and IF‐associated liver disease.^24^, ^25^ Up to 70% of PN‐associated deaths are attributable to the infection of the central venous catheter exposing this patient population to a significant health risk.^26^, ^27^, ^28^ Besides the well‐known metabolic issues and infectious risks associated with PS, patients also need to face psychological problems such as depression and anxiety due to their inability of having normal oral intake, long “hook up” times to PS restricting their mobility on day time and overnight infusions deteriorating sleeping habits.^29^

The synopsis of the clinical findings led to the experimental approach of treating the patient with the intestinotrophic growth factor teduglutide, which is currently approved and specifically indicated for the treatment of SBS patients with IF (SBS‐IF).^18^ The patients' AL‐amyloidosis with intestinal involvement showed great similarities to the clinical manifestation of SBS‐IF. Therefore, treatment with teduglutide presented a great opportunity to achieve an improved nutritional status without exposing the patient to the risks of PS. By activating the GLP‐2 receptor, teduglutide is able to induce enterocyte proliferation in the crypts and hindering enterocyte apoptosis at the top of the villi, thereby causing intestinal mucosal growth and subsequently enhancing intestinal absorptive capacity.^16^, ^17^ The results of the pivotal prospective randomized‐controlled STEPS trial and the open‐label extensions revealed that around 20% of patients on teduglutide were able to completely discontinue PS.^18^, ^30^, ^31^, ^32^ Nevertheless, subsequent retrospective cohort analyses, unbound to any study weaning protocols revealed much higher enteral autonomy rates.^33^, ^34^, ^35^, ^36^ Especially, the experience at our institution of SBS‐IF patients under teduglutide therapy showed a much‐improved outcome, with over 90% of patients achieving enteral autonomy and thereby complete independence from PS.^37^ The experiences with teduglutide at our institution, the similarity of the clinical manifestation of the patient's AL‐amyloidosis‐associated IF to SBS‐IF patients and the general good tolerability of teduglutide^38^ supported the here presented new treatment approach with teduglutide. Three months into the treatment with teduglutide as well as being closely supervised and supported by a multidisciplinary team within our institution, the patient was able to fully discontinue PN, reduce intravenous fluid intake while at the same time improving urinary output and gaining body weight. After the discontinuation of PN, the patient continued gaining weight, finally reaching a body weight of 65 kg and a total weight gain of 10 kg.

Another interesting finding of this case report is the histopathological workup of the intestinal biopsies revealing a potential explanation for the excellent clinical outcome. The patient received a colonoscopy at the initial presentation at our institution where the pathological workup in terms of a positive Congo‐Red staining of the biopsy specimens confirmed the diagnosis of intestinal involvement of the AL‐amyloidosis. After 6 months of teduglutide treatment with an excellent clinical response to the teduglutide therapy, another colonoscopy with intestinal biopsies was performed. Histopathological analysis revealed a near total disappearance of the Congo‐Red positive stained material with only remnant positive staining in single fibers of the lamina propria of the small bowel and a complete disappearance in the large bowel. As the Congo‐Red staining is highly specific for the amyloid deposits, this would mean intestinal regeneration from the amyloidosis.

Nevertheless, an explanation for the observed phenomena is yet to be awaited and needs further research. The intestinal amyloid deposits are usually observed in the stromal compartment and subsequently hinder intestinal function, while current knowledge of the functional principle of the GLP‐2/GLP‐2R axis involves the mucosal crypt‐villus axis as activation induces crypt cell proliferation and exerts anti‐apoptotic actions of the enterocytes at the tip of the villi.^16^, ^17^ Although the effector tissue is the intestinal mucosal epithelium, the actual GLP‐2R seems to be located in non‐epithelial cells such as subepithelial myofibroblasts, enteric neurons, and the enteroendocrine L‐cells with growth factors such as keratinocyte growth factor, insulin‐like growth factor 1, and ErbB subfamily of ligands transducing the intestinotrophic actions.^39^, ^40^, ^41^ The stromal location of the GLP‐2R location may hold the key to the unknown mechanism of amyloid clearance under teduglutide therapy in the here presented case.

## 4 CONCLUSION

This case report describes a new treatment approach of an amyloidosis patient with IF who was successfully treated with the GLP‐2 analogue teduglutide. The treat‐to‐target goal of enteral autonomy in such patients seems to be of utmost importance as it eases the patients of PS‐associated morbidity and mortality while enabling improved body weight and composition as well as QoL.

## AUTHOR CONTRIBUTIONS

**Clara Luhn:** Conceptualization; data curation; formal analysis; investigation; methodology; project administration; validation; visualization; writing – review and editing. **Hermine Agis:** Conceptualization; formal analysis; methodology; validation; writing – review and editing. **Elisabeth Hütterer:** Conceptualization; methodology; validation; writing – review and editing. **Ingrid Simonitsch‐Klupp:** Conceptualization; validation; writing – review and editing. **Christopher Dawoud:** Conceptualization; validation; writing – review and editing. **Anton Stift:** Conceptualization; validation; writing – review and editing. **Felix Harpain:** Conceptualization; data curation; formal analysis; methodology; project administration; resources; supervision; visualization; writing – review and editing.

## FUNDING INFORMATION

None.

## CONFLICT OF INTEREST STATEMENT

Felix Harpain reports a grant and lecture fee from Takeda, outside the submitted work. Elisabeth Hütterer and Anton Stift report lecture fees from Takeda, outside the submitted work. Clara Luhn, Hermine Agis, Ingrid Simonitsch‐Klupp, and Christopher Dawoud have indicated they have no conflict of interests to disclose.

## CONSENT

Written informed consent was obtained from the patient to publish this report in accordance with the journal's patient consent policy.

## Data Availability

Data will be made available upon reasonable request.
